# Progress of Physiology

**Published:** 1856-04

**Authors:** W. Krause

**Affiliations:** Cincinnati


### DEPARTMENT OF SELECTIONS.

### PROGRESS OF PHYSIOLOGY. TRANSLATIONS FROM GERMAN PERIODICALS FOR THE "CINCINNATI MEDICAL OBSERVER."

BY W. KRAUSE, M. D., CINCINNATI.

Russia.—Owsjannikow, of Dorpat, has written an inaugural dissertation on the microscopical structure of the spinal marrow. The most important point laid down in it, is the union of motory and sensitive nervous fibres in the spinal marrow of fishes, in one ganglianic cellule, from which cellule a single fiber ascends to the brain. Hence, should this observation be affirmed with regard to men, it will follow that this single fiber transmits the centripetal sensation, as well as the centrifugal impulse of volition, between the brain and the medulla spinalis. It is hardly necessary to mention, how simple the mechanism of reflex action may be explained hereafter. Kœlliker's skepticism, in denying the author's right to draw inferences from his observations on fishes, with respect to animals of a higher order, seems to be unfounded, as R. Wagner, of Gottingen, had previously been led to presume a similar anatomical bearing with men, by his observations on the spinal marrow of men and other mammalia. The principles of the function of the centers of the nervous system being, moreover, the same in men and fishes, there is but little reason to doubt of a similar anatomical arrangement in their nervous centers.

France.—The following are the results of E. Foltz's observations and experiments on the cerebro-spinal fluid:

1. The brain, when taken out of the calvaria, weighs about 131 grms. It is by five times lighter, when being suspended in the cerebro-spinal fluid. This accounts for the want of pressure of the brain on those parts situated at the base of the skull. The fluid, therefore, acts like a suspensory ligament.

2. The cerebro-spinal fluid diminishes, to a great extent, the violence of mechanical injuries, that might disturb the function of the nervous system, such as walking, jumping, falling, etc.

3. It regulates the circulation within the skull and the vertebral column. The anatomical properties of the cerebral vessels, favoring the arterial circulation, but predisposing to veinous congestion, the cerebro-spinal fluid prevents an abnormal distension of the walls of the veins, while favoring, at the same time, the veinous circulation, by imparting to the veins the lateral pressure of the arterial circulation.

(These propositions are sustained by ingenious experiments on dead subjects and live animals).

Germany.—Dr. Cohen, of Breslaw, mentions two cases, in which the facial nerve was found destroyed by tubercular caries of the petrous bone. There was not the slightest sensation of taste, in either case, on the corresponding half of the tongue. Both patients enjoyed there only quite indistinct feelings of touch. As the other lingual nerves were ascertained to be free from any disease, it must be admitted that the chorda tympani takes an essential part in the gustatory sensation, (Cl. Bernard), however difficult the explanation of this fact may be.

Dr. Cohen further denies Marshall Hall's assertion, that in cases of paralysis, depending on a disease of the brain, the muscular irritability is always increased. Nor are spinal paralyses always distinguished by a diminution of irritability of the paralyzed muscles.

